# Supplementary material for: Effect of Subinhibitory Concentrations of Antibiotics and Disinfectants on ISAba-Mediated Inactivation of Lipooligosaccharide Biosynthesis Genes in Acinetobacter baumannii
Source: Antibiotics (Basel). 2021 Oct 16;10(10):1259. doi: 10.3390/antibiotics10101259 (PMC8532902; doi:10.3390/antibiotics10101259)
Supplement: Supplementary file 1 [file antibiotics-10-01259-s001.zip › antibiotics-1409892-supplementary.pdf]

antibiotics

MDPI

Brief Report

Effect of Subinhibitory Concentrations of Antibiotics and Disinfectants on IS*Aba*-Mediated Inactivation of Lipooligosaccharide Biosynthesis Genes in *Acinetobacter baumannii*

Héctor Olmeda-López, Andrés Corral-Lugo † and Michael J. McConnell *,†

Intrahospital Infections Laboratory, National Centre for Microbiology, Instituto de Salud Carlos III (ISCIII), 28221 Madrid, Spain; hectorolmeda97@gmail.com (H.O.L.); acorral@isciii.es (A.C.L.)
* Correspondence: michael.mcconnell@isciii.es; Tel.: +34-918-223-896
† A.C.L. and M.J.M. contributed equally to this work and should be considered co-senior authors.

Supplementary Materials

Table S1. List of primers used in the present work.

Primer	Sequence 5′ → 3′	Target gene	GenBank ID	Source of reference
*lpxA*_Fw	TGAAGCATTAGCTCAAGTTT	*lpxA*	39519984	Moffat *et. al.*, 2010
*lpxA*_Rev	GTCAGCAAATCAATACAAGA			
*lpxC*_Fw	TGGTGAAAATCAGGCAATGA	*lxpC*	31349665	Moffat *et. al.*, 2010
*lpxC*_Rev	TGAAGATGACGTTCCTGCAA			
*lpxD*_Fw	CAAAGTATGAATACAACTTTTGAG	*lpxD*	39520015	Moffat *et. al.*, 2010
*lpxD*_Rev	TCTGCACCAGCAAACAGGAA			
IS*Aba*11_Fw_Seq	CCGGGTGATTAAACAGGTTTGT	IS*Aba*11	JF309050	This work
IS*Aba*11_Rev_Seq	TCGTGCGGTGTGTAGTCTTC			
